# Whole exome sequencing with a focus on cardiac disease-associated genes in families of sudden unexplained deaths in Yunnan, southwest of China

**DOI:** 10.1186/s12864-022-09097-0

**Published:** 2023-01-31

**Authors:** Si-Jie Wei, Jin-Liang Du, Yue-Bing Wang, Peng-Fei Qu, Lin Ma, Zhong-Chun Sun, Xue Tang, Kai Liu, Yan-Mei Xi, Sheng-Jie Nie, Peng-Lin Jia, Wu Long, Yong-Qiang Qu, Yu-Hua Li, Pu-Ping Lei

**Affiliations:** 1 Department of Forensic Medicine, Kunming Medical University, Kunming, 650500 Yunnan Province China; 2 Forensic Science Center of Jining Medical University, Jining, Shandong 272000 People’s Republic of China; 3 Yunnan Institute of Endemic Disease Control and Prevention, Dali, 671000 Yunnan Province China; 4 School of Basic Medicine, Peking University Health Science Center, Beijing, 100191 China

**Keywords:** Sudden unexplained death, Negative autopsy, Whole exome sequencing, Pathogenic variants

## Abstract

**Objectives:**

To explore the causes of sudden unexpected death (SUD) and to search for high-risk people, whole exome sequencing (WES) was performed in families with SUDs.

**Methods:**

Whole exome sequencing of 25 people from 14 SUD families were screened based on cardiac disease-associated gene variants, and their echocardiograms and electrocardiograms (ECG) were also examined. The protein function of mutated genes was predicted by SIFT, PolyPhen2 and Mutation Assessor.

**Results:**

In the group of 25 people from 14 SUD families, 49 single nucleotide variants (SNVs) of cardiac disease-associated genes were found and verified by Sanger sequencing. 29 SNVs of 14 cardiac disorder-related genes were predicted as pathogens by software. Among them, 7 SNVs carried by two or more members were found in 5 families, including SCN5A (c.3577C > T), IRX4 (c.230A > G), LDB3 (c.2104 T > G), MYH6 (c.3G > A), MYH6 (c.3928 T > C), TTN (c.80987C > T) and TTN (c.8069C > T). 25 ECGs were collected. In summary, 4 people had J-point elevation, 2 people had long QT syndrome (LQTS), 4 people had prolonged QT interval, 3 people had T-wave changes, 3 people had sinus tachycardia, 4 people had sinus bradycardia, 4 people had left side of QRS electrical axis, and 3 people had P wave broadening. Echocardiographic results showed that 1 person had atrial septal defect, 1 person had tricuspid regurgitation, and 2 people had left ventricular diastolic dysfunction.

**Conclusions:**

Of the 14 heart disease-associated genes in 14 SUDs families, there are 7 possible pathological SNVS may be associated with SUDs. Our results indicate that people with ECG abnormalities, such as prolonged QT interval, ST segment changes, T-wave change and carrying the above 7 SNVs, should be the focus of prevention of sudden death.

**Supplementary Information:**

The online version contains supplementary material available at 10.1186/s12864-022-09097-0.

## Background

The causes of sudden unexplained deaths (SUD) cannot be identified through normal medical examination methods like forensic pathological anatomy, histological examination and toxicological test. There were hundreds of SUDs that occurred in the northwest and central mountainous areas of Yunnan Province, southwest of China. As several of them occurred in the same families or same villages, these SUDs in Yunnan province had obvious characteristics of familial and spatial aggregation and caused great panic among local people.

Some studies suggested that the underlying causes of SUDs might be associated with cardiomyopathy and (or) cardiac channelopathies, such as long QT syndrome (LQTS), short QT syndrome (SQTS), Brugada syndrome (BrS), catecholaminergic polymorphic ventricular tachycardia (CPVT), arrhythmogenic right ventricular cardiomyopathy (ARVC), dilated cardiomyopathy (DCM) and hypertrophic cardiomyopathy (HCM). Besides, we have found some SNVs in some SUDs in Yunnan province [1, 2]. Owing to the characteristics of familial aggregation and village aggregation, we supposed that SUDs in Yunnan province might be related with family inheritance and their pathogenic gene variants might also exist in their relatives. Therefore, genetic tests of the living may help to identify whether pathogenic gene variants associated with SUDs are inherited from their family and then prevent the SUD in high-risk family members [3].

In order to expose potential causes of SUD and prevent their relatives from sudden deaths, we performed whole exome sequencing (WES) and echocardiogram and electrocardiogram (ECG) examinations in families with SUD.

## Results

### Family information

**Fig. 1:**
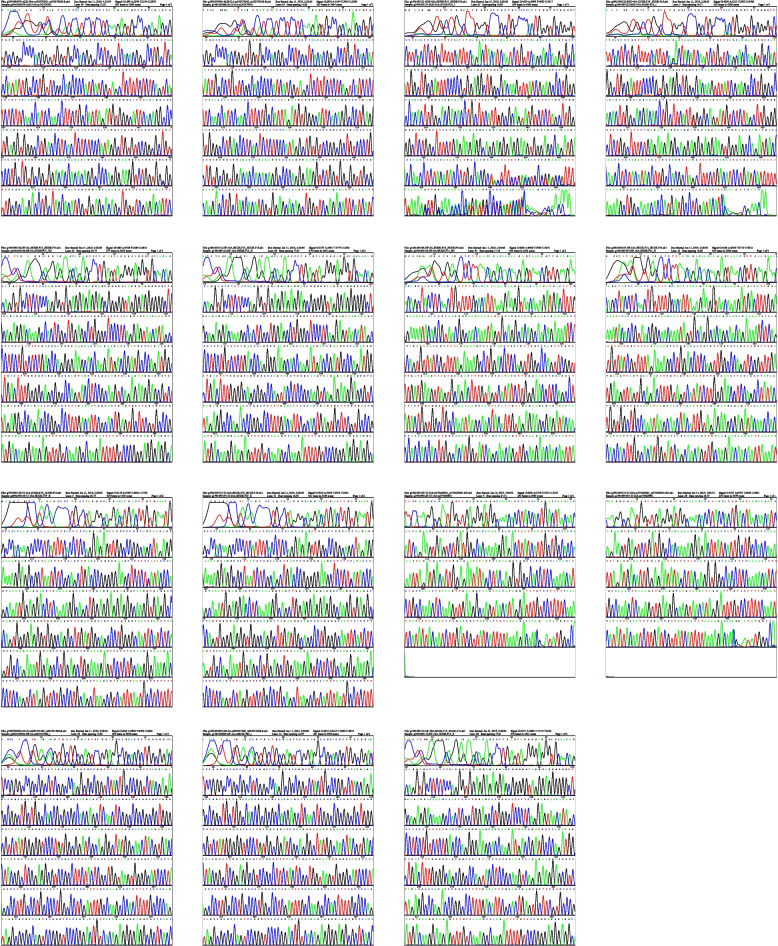
Verification results of possibly pathological SNVs by Sanger sequencing

Twenty-five members (16 males, 9 females, 4 to 74 years old) from 14 SUD families were investigated, and their information was listed in Table 1 ( see Supplementary Table 1) and the pedigree structure was drawn in Fig. 1 (see Sfig. 1).

**Table 1:** ECG data of the members from 14 SUD families for WES

NO	Age	ECG							Diagnosis
		QRS (ms)	QT/QTcB (ms)	PR (ms)	P (ms)	RR/PP (ms)	P/QRS/T (°)	HR (bpm)	
AJZ-1A	51	104	398/453	180	108	762/765	56/-31/38	78	QT interval prolonged/ Left axis deviation
ALH-19A	35	96	368/402	172	114	816/830	62/-26/34	72	J-point elevation
ALH-21A	4	68	302/435	132	70	558/480	33/84/41	125	QT interval prolonged/ sinus tachycardia
ALH-20A	3	86	318/389	132	88	648/665	43/58/29	90	-
ALH-22A	25	80	366/392	156	96	872/865	54/54/48	69	-
DP-18A	4	158	358/522	130	72	602/905	30/56/36	128	sinus tachycardia/ LQTS/
DP-2A	40	82	376/447	136	106	702/705	40/38/17	85	QT interval prolonged
DP-20A	74	74	432/519	144	106	886/880	65/64/35	87	LQTS
DP-3A	39	96	374/400	146	118	860/865	46/78/43	69	-
DP-4A	31	92	372/412	132	112	796/810	57/-25/16	74	-
HP-18A	48	98	396/418	158	102	886/895	58/-78/33	67	Left axis deviation
HP-24A	34	98	322/406	170	96	622/625	54/72/56	96	left ventricular high voltage
HP-9A	45	98	360/412	168	108	750/755	69/81/63	79	-
QS-10A	68	104	438/391	206	124	1226/1250	58/45/33	48	sinus bradycardia/ first degree atrioventricular block/ p-wave prolonged/ J-point elevation
SGZ-42A	63	84	394/431	160	116	922/830	70/28/63	72	left ventricular high voltage/ T-wave change
SGZ-43A	11	94	382/415	136	96	872/845	53/-54/41	71	Left axis deviation
SJ-6A	46	102	382/446	132	110	732/730	67/68/37	82	QT interval prolonged
TJ-15A	42	98	370/418	172	108	766/775	65/53/43	77	-
TJ-22A	64	94	408/427	182	138	886/905	75/33/60		p-wave prolonged
TJ-25A	66	98	352/435	174	106	644/650	79/82/82	92	J-point elevation
TJ-30A	55	88	302/403	180	146	554/560	67/29/47	107	sinus tachycardia/ P-wave prolonged/ T-wave lowflat
TJ-8A	59	92	422/428	174	108	974/965	80/67/60	60	-
GT-6A	53	78	434/418	154	118	1086/1070	54/39/28	56	sinus bradycardia/ T-wave lowflat
GT-35A	24	98	396/388	162	114	1020/1030	51/-57/-25	58	sinus bradycardia/Left axis deviation/J-point elevation
GT-37A	20	104	408/397	170	94	1008/1050	29/94/75	57	sinus bradycardia

^a^: The same English number means same family

### ECG data

Twenty-five ECGs were collected (all ECG data shown in Table 1). In summary, 4 people had J-point elevation, 2 people had LQTS, 4 people had prolonged QT interval, 3 people had T-wave change, 3 people had sinus tachycardia, 4 people had sinus bradycardia, 4 people had left side of QRS electrical axis, and 3 people had P wave broadening.

### Echocardiogram examination

Echocardiogram results showed that one 11-year-old boy (SGZ-43A) had atrial septal defect, 1 person had tricuspid regurgitation, and 2 people had left ventricular diastolic dysfunction.

### Whole exome sequencing

WES was performed in 25 samples from 14 SUD families. 58.97 Mb of the target area was captured by the chip. The clean reads of each sample were compared with the human reference genome sequence (GRCh37/HG19), and the average reads of 99.52% were compared with the reference genome. After removing duplicate reads, an average of 434,508,841 effective reads were obtained (i.e. 21,605.59 Mb effective bases). In the effective bases, 45.33% of the value of capture specificity was specified in the target region. The average sequencing depth of the target region was about 165.71 × . On average, 99.88% of the target region in each sample was covered by at least 1 read, and 97.78% of the target region was covered by at least 10 reads.

In all samples, 284,082 SNPs/SNVs (single nucleotide polymorphisms/variants) were found, 90.03% of which appeared in the dbSNP database and 89.76% in the 1000 genome project database. There are 21,012 newly discovered SNPs/SNVs. In the overall SNPs/SNVs, there are 26,637 synonymous variants and 28,035 missense variants in the coding region, among which 55 SNVs are termination codon loss, 309 SNVs are termination codon gain, 60 SNVs are initiation codon loss, and 239 SNVs are splicing region variants (splicing receptor or splicing donor variants). 49 SNVs were found in 38 cardiac disease-associated genes.

46,352 InDels (insert and deletion polymorphisms/variants) were found in all samples, 59.39% of which were in the dbSNP database and 48.10% in the 1000 Genomes Project database. There are 17,246 new InDels discoveries. Overall, there are 97 frameshift variants in the coding region, 14 have termination codon loss, 6 have initiation codon loss, and 91 have splicing region variants. 19 genes of heart disease-associated susceptibility gene InDels, and a total of 38 SNVs were found.

### Variant validation

We annotated all the SNVs/ InDels, that meet the quality control standards in WES by using the ANNOVAR software and saved the results in an EXCEL spreadsheet. To exclude the pseudo variants, all variants found in the samples were sequenced by using the conventional Sanger sequencing. Among all SNVs, 29 cardiac disease-associated gene variants were further aggregated and validated (see Supplementary Table 2). In the screening of InDels genes, no pathogenic variants were found.

Among 29 cardiac disease-associated gene variants, seven possibly pathogenic SNVs were found in 5 families: ① In family 2, ALH-19A with J-point elevation and ALH-21A with QT interval prolongation/ sinus tachycardia co-carry SCN5A (c.3577C > T); ② In family 4, IRX4 (c.230A > G) is a common SNV in DP-2A with QT interval prolongation, DP-4A and DP-18A with QT interval prolongation/ sinus tachycardia; ③ In family 6, MYH6 (c.3G > A), and TTN (c.80987C > T) are SNVs carried by HP-9A and HP-18A with Left axis deviation; ④ In family 9, LDB3 (c.2104 T > G) was found in SGZ-42A with left ventricular high voltage/T-wave change and SGZ-43A with left axis deviation; ⑤ In family 11, MYH6 (c.3928 T > C) and TTN (c.8069 C > T) were shared by TJ-15A and TJ-25A with J-point elevation (see Fig. 1).

## Discussion

For nearly half a century, forensic and clinical pathologists have been struggling to find the cause of SUDs in Yunnan province in China, and put forth some theories including enterovirus infection, fulminant myocarditis and wild mushrooms poisoning. However, these causes can only explain some SUD cases in Yunnan province [4–6]. Despite the intervention and prevention of the above-mentioned factors, there is still cluster obesity in Yunnan Province, which suggests that obesity in Yunnan Province may be related to other factors. Owing to the inconvenient transportation, the phenomenon of intermarriage within several neighbor villages in the epidemic area of SUDs has been existing more than hundreds of years, without genetic communication with other areas. Therefore, some scholars supposed that pathogenic gene variants may lie in these SUD families [7].

In order to make sure whether these SUDs in Yunnan province were related to heritable genovariation of cardiac diseases, we performed WES, echocardiogram and ECG examinations in the family members with SUDs. Through Sanger verification, we obtained 29 SNVs of 14 genes. Among them, 7 SNVs carried by two or more members were found in 5 families, including SCN5A (c.3577C > T), IRX4 (c.230A > G), LDB3 (c.2104 T > G), MYH6 (c.3G > A), MYH6 (c.3928 T > C), TTN (c.80987C > T) and TTN (c.8069C > T).

The rate of TTN (titin) vatiants was highest, reaching 44.82%, and 13 out of 25 people had TTN SNVs. TTN, coding a scaffold protein aiding in myofibrillar assembly during myogenesis, has been reported to participate in the biological processes of cardiac myocyte hypertrophy, myofibroblastic development, cardiomyocyte differentiation and myocardial contraction. Previous research showed that TTN gene variants were associated with various cardiomyopathies and a range of skeletal muscle diseases. It was found that TTN variations are mainly related to DCM in Caucasians [8]. TTN interacts with other ion channel proteins, leading to sick sinus syndrome (SSS) when variations occur [9]. However, in our study, no lethal pathological changes were observed by echocardiogram. Thus, the TTN variations still could not be considered as pathogenic.

There were 4 cases of MYH6 (myosin heavy chain) variations. MYH6 encodes alpha-myosin heavy chain expressed in the developing atria, which affects the binding of the heavy chain to its regulatory light chain [10]. MYH6 plays a pivotal role in the development of the atrial septum, and MYH6 variation is associated with atrial septal defect (ASD) [9]. In addition, MYH6 is the gene causing human DCM and HCM. HCM is the leading cause of sudden death in the athletic field and the most common cause of nonviolent sudden death in young adults [11]. In our study, although one 11-year-old boy (SGZ-43A) had atrial septal defect, he did not have MYH6 variants. Therefore, the MYH6 variant also could not be regarded as pathogenic.

We found that HP-9A and HP-18A had the same SNVs of MYH6 (c.3G > A), TTN (c.80987C > T). TJ -15a and TJ -25a had the same SNVs of MYH6 (c.3928 T > c), and TTN (c.8069C > T). HP-9A and HP-18A are brothers, and the father of HP-9A and HP-18A is SUD. HP-9A and HP-18A have the same genetic relationship with their father, and these SNV and InDel mutation sites may be derived from the same variants. The protein function prediction of these SNVs showed high pathogenicity, ECG results showed that the HP-9A and HP-18A electrical axes were leftward, but the color Doppler ultrasound showed no obvious abnormalities. So the MYH6 (c.3G > A) and TTN (c.80987C > T) variants may be related to the sudden death of the father of HP-9A and HP-18A. TJ -22a and TJ-25A are husband and wife, TJ-15A is their son, TJ-22A and TJ-25A's son (the younger brother of TJ-15A) is SUD, TJ -15A, TJ-22A and since TJ-25A have a genetic relationship, these SNVs mutation sites may be derived from the same variant. The protein function predictions of these SNVs showed strong pathogenicity. ECG results showed that the J point of TJ-15A and TJ-25A were elevated, and the color Doppler ultrasound results showed that the left ventricular diastolic function of TJ-15A and TJ-25A was reduced. MYH6 (c.3928 T > C) and TTN (c.8069C > T) variants may be related to the sudden death of the offspring of TJ-22A.

IRX4 (Iroquois homeobox 4) variants were present in 4 patients. Research shows that IRX4 variants can lead to the suppression of myosin heavy chain gene expression, resulting in abnormal gene expression and ventricular cardiac hypertrophy, closely related to heart development [12–14], IRX4 variants can cause cardiac insufficiency and cardiomyopathy of congenital heart disease, especially the occurrence of ventricular septal defect with potential influence [15]. We found that DP-2A, DP-4A and DP-18A had the same SNV variants. IRX4 (c.230A > G) variants were discovered for the first time in people in southern China. DP-2A is DP-4A’s aunt, DP-2A's father and sister (DP-4A's mother and DP-18A's grandmother) are SUDs, DP-2A and DP-4A have a genetic relationship with DP-18A's grandmother, and this SNV may be derived from the same variant. The prediction of protein function of this SNV showed high pathogenicity. ECG showed prolonged QT interval in DP-2A, QT prolongation / sinus tachycardia in DP-4A and DP-18A, and no abnormal results were found by color Doppler ultrasound. IRX4 (c 0.230A > G) variant may be related to the sudden death of DP-4A mother and grandfather.

There are 2 cases of LDB3 (lim domain binding 3) variant, which encodes for cipher (mouse)/zasp (human), a cytoskeletal protein. This protein is a crucial component of the sarcomeric z-disks in binding critical proteins. LDB3 variant can cause cardiac dysfunction such as myofibrillar myopathy, DCM, arrhythmia and cardiomyopathy [16], as well as arrhythmia right ventricular dysplasia (ARVD) [17]. In our study, we found that SGZ-42A and SGZ-43A had the same SNV, LDB3 (c.2104t > G). SGZ -42A is SGZ-43A’s grandfather. Both the wife and two daughters of SGZ-42A are SUDs. So, this LDB3 SNV may be derived from the same variant. The protein function prediction of LDB3 SNV showed high pathogenicity. The ECG results showed that there were left ventricular hypertension/T wave in SGZ-42A, left deviation of electrical axis in SGZ-43A, and the color Doppler ultrasound results showed that SGZ-43A has a congenital heart disease (atrial septal defect). Therefore, the variant of LDB3 (c. 2104) may be related to the sudden death of SGZ-42A's wife and two daughters.

In addition to above four variant genes that can cause structural changes in the heart, we also found a SNV of an ion channel-related gene. The SCN5A gene encodes an alpha subunit of I_Na_ associated sodium channel (Na_V_1.5). Hundreds of SCN5A variants have been reported leading to abnormal function of sodium ion channels and myocardial repolarization disorder [18, 19], including BrS, LQTS, SSS, etc. Abnormal sodium ion channels caused by SCN5A gene variant can be activated by high temperature, so BrS has a higher probability of incidence in high temperature regions which may help to explain the summer aggregation of SUD in some epidemic areas [20]. The onset of BrS is insidious, and the main changes of ECG are ST segments: J point elevation and ST segment dome-shaped or saddle-shaped elevation. In this study, 4 people had J point elevation, but no other changes of ST segment were found. We cannot identify or exclude the diagnosis of BrS by J point elevation alone. However, J point elevation is a sign of early repolarization, and some scholars call it early repolarization syndrome [21]. To be sure, when a family has a history of sudden death, early repolarization is considered valuable, or a harbinger of BrS. Variation of SCN5A (c.3577C > T) was found in ALH-19A, with elevated J point. ALH-21A, the son of ALH-19A, also carries SCN5A variation. He has sinus tachycardia and T wave inversion in V1 ~ V3 leads. Without horizontal and vertical comparison, it was not confirmed that SCN5A (c.3577C > T) was the cause of his abnormal ECGs. Among the subjects with J-point elevation, the age span was large, and two of them were over 60 years old. No abnormality was found by color Doppler echocardiography. Whether they had coronary heart disease or in hyperacute stage of acute coronary syndrome was not yet determined.

Nine residual variant genes were found only in one case, including NPHP4, CSRP3, SYNPO2L, MCTP2, LAMA4, LMNA, ACTN2, DMPK, ANK2. CSRP3 (cysteine and glycine-rich protein 3) encodes muscle LIM protein (MLP), which is the pathogenic gene of HCM and DCM [22, 23]; SYNPO2L (synaptopodin 2 like) is a gene encoding actin-related protein, which can play a role in regulating the shape of actin, and its gene variant can cause AF [24]; MCTP2 (multiple C2 and transmembrane domain containing 2) may play a role in the development of the cardiac outflow tract, and its variant can lead to cardiac arrest [25]; LAMA4 (laminin 4 subunit), which mainly encodes laminin and is involved in cell adhesion, differentiation, migration, signal transduction, neurite outward growth and metastasis, is the pathogenic gene of DCM [26]; LMNA (lamin A) is the gene encoding nuclear membrane protein, and it is the pathogenic gene of ARVC and DCM [27, 28]; ACTN2 (α-actin-2) encoding α-actin is the pathogenic gene of HCM [29]; DMPK is a gene encoding serine-threonine kinase, which acts on L-type calcium channels, and its variants can cause left ventricular hypertrabeculation (LVHT) [30]; ANK2 is the pathogenic gene of LQTS. All protein functions of these variant genes are predicted to be highly pathogenic by software.

At present, the lack of gene-qualified, biological samples of the deceased, the screening of cardiac disease-associated genes in SUD family could help to reveal the etiology of SUD. Although screening strategies and protein prediction tools could help explain the pathogenicity of variants, the pathological and functional consequences of variants are often unclear and require further functional studies. Accurate interpretation is more difficult in complex multifactorial phenotypes, such as SUD without any significant morphological abnormalities, and lethality may be triggered by different events such as personal or environmental stress, medication or physical activity. In addition, the analysis of family co-separation is crucial to verify possible pathogenic effects of observed variation. Because some family members were not present or refused to take part in our research, genetic information of all members could not be provided, and analysis of co-separation could not be completed.

Currently, there are several effective prevention measures for conditions leading to heart attacks, such as lifestyle amelioration, pathogenic block, medication and implantable defibrillators, to prevent another sudden death in SUD family. Through screening of susceptibility genes, SUD types in different regions and families could be identified that may provide guidance for its prevention and treatment.

## Conclusions

Of the 14 heart disease-associated genes in 14 SUDs families, there are 7 possible pathological SNVS may be associated with SUDs. Our results indicate that people with ECG abnormalities, such as prolonged QT interval, ST segment changes, T-wave change and carrying the above 7 SNVs, should be the focus of further study for prevention of sudden death.

## Materials and methods

### Study population

Study populations were selected from villages with a high incidence of SUD in Chuxiong, Dali, Lijiang and Lincang located in the central and northwestern regions of Yunnan province, southwest of China. Twenty-five (25) people from 14 families with SUD histories were immediate family members of SUDs. All SUDs were determined by investigations and examinations of the Endemic Disease Prevention and Treatment Institute in Yunnan province.

### Electrocardiogram (ECG) examination

ECG (MAC1200ST, GE, USA) examinations were performed on each subject, and ECG diagnoses were carried out independently by 2 cardiologists.

### Echocardiography examination

Terasont3000 ultrasound system (Taratech, Burlington, MA 01,803, USA) was used. The probe frequency was 2–4 MHz and the detection depth was 16–20 cm. All of patients were operated by two experienced sonographers. Assist the patient in supine or left-side supine position, guide them to adjust their respiratory state, take the long axis section of the left edge of the sternum for M-type sampling, and observe the patient's ventricular wall thickness, internal diameter and size of each atrium, closure status, and valve shape.

### DNA extraction and quantification

Genomic DNA was obtained from peripheral venous blood preserved in EDTA tube. All DNA extractions were performed by using the TIANamp blood DNA kit, DP318 (Tiangen, Beijing, China), according to manufacturer’s recommendations. All DNA quantities were determined with a Qubit 1.0 fluorometric quantification device (ThermoFisher, Waltham, MA).

### Whole exome sequencing

Exome capture, sequencing as well as sequence alignment and variant calling were performed at BGI (Beijing Genomics Institute) Medical Laboratory in Wuhan, China. Sequencing was done on the Illumina HiSeq2000 platform (Illumina Inc., San Diego, CA) generating 2 × 100 bp paired end reads. A filter was set so that one sample was required to have at least 96% of exome covered at ≥ 100 × read depth. Alignment of sequence reads, indexing of the reference genome (hg19), and variant calling and annotation were carried out with a pipeline based on BWA, Samtools, Picard and Annovar.

### Data analysis

An annotated list of all SNVs/ InDels that meets quality control standards was provided by an Excel (Microsoft, Redmond, WA) spreadsheet (see Sfig 2). Following WES and variant annotation, variant filtration involving the exclusion of all non-coding regions and synonymous variants (i.e. DNA nucleotide alteration amino acid sequence of the protein) and gene-specific analysis of the 289 genes linked to heart diseases were performed to identify potential pathogenic variant(s) [31–33]. To consider a possible pathogenic variant associated with SUD, the minor allele frequency (MAF) of any variant discovered had to be less than 0.01 in the public available exome database 1,000 Genome Project.

### Variant confirmation

All possible pathogenic variant found in the relatives of SUD cases were confirmed using standard polymerase chain reaction (PCR) and Sanger DNA sequencing methods. PCR primers, conditions, sequencing methods and variant sequences are available upon request (see Supplementary Table 3). Sequences were output in BAM format. Raw data were deposited in SRA (PRJNA863403). 

## Supplementary Information

**Additional file 1:** **Figure 1.** The pedigree structures with SUD for WES.

**Additional file 2:** **Supplementary Figure 2.** all SNVs InDels that meets quality control standards.

**Additional file 3:** **Table 1.** Information of the persons from SUD families for WES.

**Additional file 4:** **Table 2.** WES results of susceptible SNVs with a focus on cardiac disease-associated genes in SUD families.

**Additional file 5. **

## Data Availability

All data generated or analysed during this study are included in this published article and its supplementary information files.

## Abbreviations

SUD: Sudden unexpected death
WES: Whole exome sequencing
ECG: Electrocardiograms
SNV: Single nucleotide variant
SNP: Single nucleotide polymorphisms
InDels: Insert and deletion polymorphisms/variants
LQTS: Long QT syndrome
BrS: Brugada syndrome
ARVC: Arrhythmogenic right ventricular cardiomyopathy
DCM: Dilated cardiomyopathy
HCM: Hypertrophic cardiomyopathy
SSS: Sick sinus syndrome
TTN: Titin
MYH6: Myosin heavy chain
IRX4: Iroquois homeobox 4
LDB3: Lim domain binding 3

## References

[1] Li LJ, Wang YB, Qu PF, Ma L, Liu K, Yang L (2020). Genetic analysis of Yunnan sudden unexplained death by whole genome sequencing in Southwest of China. J Forensic Leg Med.

[2] Liu K, Wang YB, Nie SJ, Ma L, Dai QY, Tang X (2020). Study of Yunnan sudden unexplained death in Han population and cardiac disease associated genetic variants. Chin J Forensic Med.

[3] Ackerman MJ, Priori SG, Willems S, Berul C, Brugada R, Calkins H (2011). HRS/EHRA Expert Consensus Statement on the State of Genetic Testing for the Channelopathies and Cardiomyopathies. Europace.

[4] Shi GQ, Zhang J, Huang WL, Yang T, Ye SD, Sun XD (2006). Retrospective study on 116 unexpected sudden cardiac deaths in Yunnan. China Chin J Epidemiol.

[5] Li ZX, Huang WL, Zhao S, Yang L, Yang LP, Ma L (2005). Report of pathologic research of Yunnan endemic fulminant myocarditis. Chin J Endemiol.

[6] Yang L, Huang WL, Li ZX, Li JG, Zhao S, Wang YB (2008). Retrospective Research on Unexpected Sudden Cardiac Death in Yunnan From 1975 to 2004. J Prev Med Inf.

[7] Jia PL, Wang YB, Fu H, Huang WL, Zhong SR, Ma L (2018). Postmortem Analysis of 4 Mutation Hotspots of KCNQ1, KCNH2, and SCN5A Genes in Sudden Unexplained Death in Southwest of China. Am J ForensicMed Pathol.

[8] Vikhorev PG, Smoktunowicz N, Munster AB, Copeland O, Kostin S, Montgiraud C (2017). Abnormal contractility in human heart myofibrils from patients with dilated cardiomyopathy due to mutations in TTN and contractile protein genes. Sci Rep.

[9] Zhu YB, Luo JW, Jiang F, Liu G (2018). Genetic analysis of sick sinus syndrome in a family harboring compound CACNA1C and TTN mutations. Mol Med Rep.

[10] Patrice Bouvagnet JL, Francoise P, Claude D, Jean JL (1984). Fiber Types and Myosin Types in Human Atrial and Ventricular Myocardium An Anatomical Description. Circulation Res.

[11] Maron BJ, Doerer JJ, Haas TS, Tierney DM, Mueller FO (2009). Sudden deaths in young competitive athletes: analysis of 1866 deaths in the United States, 1980–2006. Circulation.

[12] Molck MC, Simioni M, Paiva VT, Sgardioli IC, Paoli MF, Souza J (2017). Genomic imbalances in syndromic congenital heart disease. J Pediatr (Rio J).

[13] Wu SP, Cheng CM, Lanz RB, Wang T, Respress JL, Ather S (2013). Atrial identity is determined by a COUP-TFII regulatory network. Dev Cell.

[14] Nelson DO, Lalit PA, Biermann M, Markandeya YS, Capes DL, Addesso L (2016). Irx4 Marks a Multipotent, Ventricular-Specific Progenitor Cell. Stem Cells.

[15] Cheng Z, Wang J, Su D, Pan H, Huang G, Li X (2011). Two novel mutations of the IRX4 gene in patients with congenital heart disease. Hum Genet.

[16] Li Z, Ai T, Samani K, Xi Y, Tzeng HP, Xie M (2010). A ZASP missense mutation, S196L, leads to cytoskeletal and electrical abnormalities in a mouse model of cardiomyopathy. Circ Arrhythm Electrophysiol.

[17] Moric-Janiszewska E, Markiewicz-Loskot G (2007). Review on the genetics of arrhythmogenic right ventricular dysplasia. Europace.

[18] Nagase S, Kusano KF, Morita H, Nishii N, Banba K, Watanabe A (2008). Longer repolarization in the epicardium at the right ventricular outflow tract causes type 1 electrocardiogram in patients with Brugada syndrome. J Am Coll Cardiol.

[19] Mullally J, Goldenberg I, Moss AJ, Lopes CM, Ackerman MJ, Zareba W (2013). Risk of life-threatening cardiac events among patients with long QT syndrome and multiple mutations. Heart Rhythm.

[20] Hong Q, Xiong QM (2010). Clinical and Molecular Biology Progress of Brugada Syndrome. Adv Cardiovasc Dis.

[21] Haïssaguerre M, Derval N, Sacher F, Jesel L, Deisenhofer I, de Roy L (2008). Sudden Cardiac Arrest Associated with Early Repolarization. N Engl J Med.

[22] Mehroz Ehsana MK, Charlotte H, Arash Y, Julia B, Mohamed B, Kirandeep G (2018). Mutant Muscle LIM Protein C58G causes cardiomyopathy through protein depletion. J Mol Cell Cardiol.

[23] Ingles J, Goldstein J, Thaxton C, Caleshu C, Corty EW, Crowley SB (2019). Evaluating the Clinical Validity of Hypertrophic Cardiomyopathy Genes. Circ Genom Precis Med.

[24] Lubitz SA, Brody JA, Bihlmeyer NA, Roselli C, Weng LC, Christophersen IE (2016). Whole Exome Sequencing in Atrial Fibrillation. PLoS Genet.

[25] Aouizerat BE, Vittinghoff E, Musone SL, Pawlikowska L, Kwok PY, Olgin JE, Tseng ZH (2011). GWAS for discovery and replication of genetic loci associated with sudden cardiac arrest in patients with coronary artery disease. BMC Cardiovasc Disord.

[26] Abdallah AM, Carlus SJ, Al-Mazroea AH, Alluqmani M, Almohammadi Y, Bhuiyan ZA, Al-Harbi KM (2019). Digenic Inheritance of LAMA4 and MYH7 Mutations in Patient with Infantile Dilated Cardiomyopathy. Medicina (Kaunas).

[27] Sirisha MC, Scot JM, Cristian C, Xin H, Matthew JR, Mary S (2019). Genomic Reorganization of Lamin-Associated Domains in Cardiac Myocytes is Associated with Differential Gene Expression and DNA Methylation in Human Dilated Cardiomyopathy. Circ Res.

[28] Ollila L, Nikus K, Holmstrom M, Jalanko M, Jurkko R, Kaartinen M (2017). Clinical disease presentation and ECG characteristics of LMNA mutation carriers. Open Heart.

[29] Haywood NJ, Wolny M, Rogers B, Trinh CH, Shuping Y, Edwards TA, Peckham M (2016). Hypertrophic cardiomyopathy mutations in the calponin-homology domain of ACTN2 affect actin binding and cardiomyocyte Z-disc incorporation. Biochem J.

[30] Finsterer J (2009). Cardiogenetics, neurogenetics, and pathogenetics of left ventricular hypertrabeculation/noncompaction. Pediatr Cardiol.

[31] Christiansen SL, Hertz CL, Ferrero-Miliani L, Dahl M, Weeke PE, LuCamp (2016). Genetic investigation of 100 heart genes in sudden unexplained death victims in a forensic setting. Eur J Hum Genet.

[32] Stallmeyer B, Dittmann S, Seebohm G, Müller J, Schulze-Bahr E (2017). Molecular genetic diagnostics for ventricular arrhythmias and sudden cardiac death syndromes. Herz.

[33] Trenkwalder T, Schunkert H, Reinhard W (2020). Sinnvolle Diagnostik: Genetik. Herz.

