# Supplementary material for: Whole exome sequencing with a focus on cardiac disease-associated genes in families of sudden unexplained deaths in Yunnan, southwest of China
Source: BMC Genomics. 2023 Jan 31;24:57. doi: 10.1186/s12864-022-09097-0 (PMC9890689; doi:10.1186/s12864-022-09097-0)
Supplement: Supplementary file 1 — Additional file 1: Figure 1. The pedigree structures with SUD for WES. [file 12864_2022_9097_MOESM1_ESM.pdf]

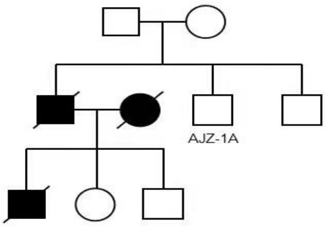

Family 1

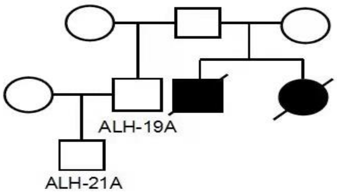

Family 2

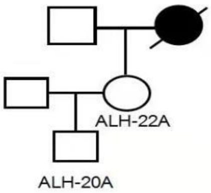

Family3

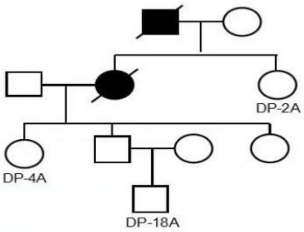

Family 4

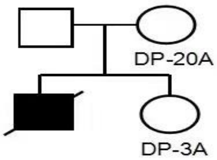

Family 5

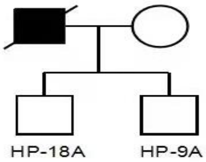

Family6

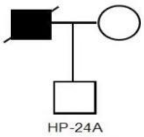

Family 7

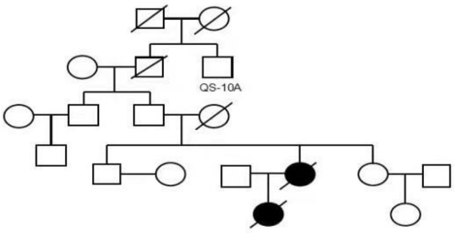

Family 8

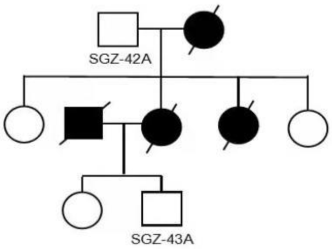

Family9

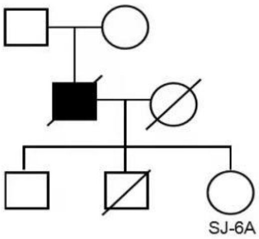

Family10

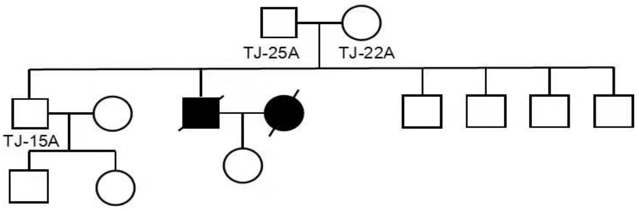

Family11

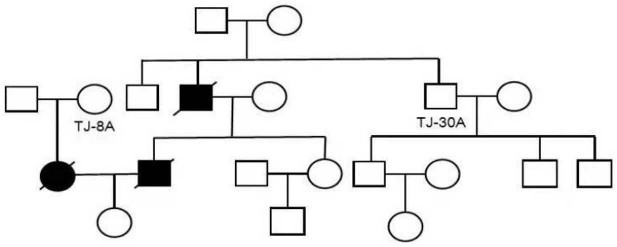

Family12

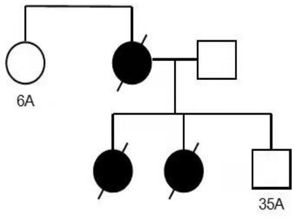

Family13

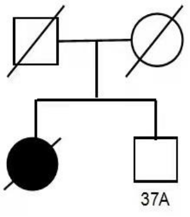

Family14

Figure 1. The pedigree structures with SUD for WES

Squares: male; Circles: female; Diagonal line: deceased individual; SUD individuals are indicated with black filled symbols, whereas members died of lethal diseases are indicated by blank symbols and diagonal lines.
