# Supplementary material for: Whole exome sequencing with a focus on cardiac disease-associated genes in families of sudden unexplained deaths in Yunnan, southwest of China
Source: BMC Genomics. 2023 Jan 31;24:57. doi: 10.1186/s12864-022-09097-0 (PMC9890689; doi:10.1186/s12864-022-09097-0)
Supplement: Supplementary file 3 — Additional file 3: Table 1. Information of the persons from SUD families for WES. [file 12864_2022_9097_MOESM3_ESM.pdf]

Table 1. Information of the persons from SUD families for WES

NO.	Family Number*	Gender	Age (year)	NO.	Family number*	Gender	Age (year)
AJZ-1A	1	male	51	HP-9A	8	male	45
ALH-19A	2	male	35	HP-18A	8	female	48
ALH-21A	2	male	4	HP-24A	9	male	34
ALH-20A	3	male	3	QS-10A	10	male	68
ALH-22A	3	female	25	SGZ-42A	11	male	65
DP-2A	4	female	40	SGZ-43A	11	male	11
DP-4A	4	female	31	SJ-6A	12	female	46
DP-18A	4	male	5	TJ-15A	13	male	42
DP-3A	5	female	39	TJ-22A	13	female	64
DP-20A	5	female	74	TJ-25A	13	male	66
GT-6A	6	male	53	TJ-8A	14	female	59
GT-35A	6	male	24	TJ-30A	14	male	55
GT-37A	7	male	20				

*: The same number means same family.
