# Supplementary material for: Whole exome sequencing with a focus on cardiac disease-associated genes in families of sudden unexplained deaths in Yunnan, southwest of China
Source: BMC Genomics. 2023 Jan 31;24:57. doi: 10.1186/s12864-022-09097-0 (PMC9890689; doi:10.1186/s12864-022-09097-0)
Supplement: Supplementary file 4 — Additional file 4: Table 2. WES results of susceptible SNVs with a focus on cardiac disease-associated genes in SUD families. [file 12864_2022_9097_MOESM4_ESM.pdf]

Table 2. WES results of susceptible SNVs with a focus on cardiac disease-associated genes in SUD families

Gene	Transcript	1000G_ALL*	1000G_EAS**	dbSNP141	SIFT***	PolyPhen2#	Mutation Assessor##	Samples
IRX4	NM_001278633.1:p.Tyr77Cys/c.230A>G	0.000599042	0.002	rs201931586	0.04（D）	0.995（D）	2.17（M）	DP-2A, DP-4A, DP-18A, HP-24A
LDB3	NM_001171610.1:p.Cys702Gly/c.2104T>G	_	_	_	0（D）	0.998（D）	4.475（H）	SGZ-42A, SGZ-43A
MYH6	NM_002471.3:p.Tyr1310His/c.3928T>C	_	_	_	0.02（D）	0.974（D）	2.175（M）	TJ-15A, TJ-25A
MYH6	NM_002471.3:p.Met1?/c.3G>A	_	_	_	0.01（D）	0.968（D）	_	HP-9A, HP-18A
SCN5A	NM_001099404.1:p.Arg1193Trp/c.3577C>T	0.000399361	0.001	rs192379242	0(D)	0.964(D)	1.93(M)	ALH-19A, ALH-21A
SCN5A	NM_001099404.1:p.Val1279Ile/c.3835G>A	_	_	rs199473341	0.02（D）	0.995（D）	3.375（M）	AJZ-1A
ACTN2	NM_001103.3:p.Asp673Ala/c.2018A>C	_	_	_	0.03（D）	0.995（D）	2.485（M）	ALH-19A
ANK2	NM_001148.4:p.Asp3707Asn/c.11119G>A	0.000399361	0	rs199549660	0.03（D）	0.551（P）	1.78（L）	TJ-8A
CSRP3	NM_003476.4:p.Gly6Arg/c.16G>A	0.000599042	0.002	rs185980145	0.04（D）	0.998（D）	2.65（M）	SGZ-43A
DMPK	NM_001081562.2:p.Pro605Leu/c.1814C>T	_	_	_	0.03（D）	0.998（D）	_	GT-37A
LAMA4	NM_001105206.2:p.Leu492His/c.1475T>A	0.00259585	0.0099	rs3752579	0（D）	0.842（P）	1.445（L）	DP-3A
LMNA	NM_170707.3:p.Arg527Cys/c.1579C>T	_	_	rs57318642	0（D）	0.953（D）	2.56（M）	GT-6A
MCTP2	NM_018349.3:p.Pro398Leu/c.1193C>T	_	_	_	0（D）	0.999（D）	2.95（M）	ALH-20A
SLC8A1	NM_021097.2:p.Ala327Thr/c.979G>A	_	_	_	0.01（D）	0.965（D）	2.105（M）	QS-10A
SLC8A1	NM_021097.2:p.Ser70Tyr/c.209C>A	_	_	_	0（D）	0.991（D）	2.675（M）	GT-37A
SYNPO2L	NM_001114133.2:p.Glu26Lys/c.76G>A	_	_	_	0.01（D）	0.7（P）	3.02（M）	GT-6A
TTN	NM_001267550.2:p.Pro26996Leu/c.80987C>T	_	_	_	0（D）	0.842（P）	3.46（M）	HP-9A, HP-18A
TTN	NM_001267550.2:p.Thr2690Ile/c.8069C>T	0.00179712	0.0079	rs374620001	0（D）	0.976（D）	2.495（M）	HP-24A, TJ-15A, TJ-25A
TTN	NM_001267550.2:p.Arg29578His/c.88733G>A	0.00139776	0.005	rs374147064	0（D）	0.999（D）	2.175（M）	SGZ-42A, TJ-8A
TTN	NM_001267550.2:p.Glu32685Gly/c.98054A>G	_	_	_	0（D）	0.998（D）	2.805（M）	GT-6A
TTN	NM_001267550.2:p.Asp16118Gly/c.48353A>G	0.000399361	0.002	rs376273101	0（D）	0.999（D）	0.41（N）	GT-6A

TTN	NM_001267550.2:p.Gly7677Arg/c.23029G>A	0.000399361	0.002	rs367826445	0（D）	0.993（D）	4.685（H）	GT-6A
TTN	NM_001267550.2:p.Gly16881Ala/c.50642G>C	0.000199681	0.001	rs201302681	0（D）	0.999（D）	2.24（M）	GT-37A
TTN	NM_001267550.2:p.Arg18050Cys/c.54148C>T	0.00139776	0.0069	rs55734111	0（D）	0.95（D）	1.215（L）	GT-35A
TTN	NM_001267550.2:p.Val28895Met/c.86683G>A	0.00299521	0	rs201290358	0（D）	0.568（P）	2.345（M）	QS-10A
TTN	NM_001267550.2:p.Arg15731His/c.47192G>A	0.000399361	0	rs373613871	0（D）	0.999（D）	3.625（H）	QS-10A
TTN	NM_001267550.2:p.Pro6384Thr/c.19150C>A	0.00159744	0.003	rs72648953	0（D）	0.997（D）	3.215（M）	ALH-20A
TTN	NM_001267550.2:p.Tyr17441Asp/c.52321T>G	_	_	_	0（D）	0.999（D）	4.37（H）	HP-24A
TTN	NM_001267550.2:p.Pro13673Leu/c.41018C>T	_	_	_	0（D）	0.47（P）	1.955（M）	DP-20A

*: The allele frequency of the mutated base at the site of the mutation was found in all population groups that gave data on the 1000-person genome project. (n=2504).

**: The allele frequency of the mutated base at the mutation site in an East Asian population with data from the 1000-person genome project. Samples were derived from the sum (n=405) of CDX (Chinese Dai in Xishuanbanna, China, n=93) , CHB (Han Chinese in Beijing, China, n=103) , CHS (Southern Han Chinese, n=105) , and JPT(Japanese in Tokyo, Japan, n=104).

***: Indicating the effect of this variation on the protein sequence. The comma before and after the SIFT _score and SIFT _pred：SIFT _score is SIFT scores, the smaller the score is, the more likely it is "harmful ", indicating that the SNP is likely to cause changes in protein structure or function. SIFT _pred is the predicted result with a value of T or D. When this variation affects multiple protein sequences at the same time, there is a SIFT value for each protein sequence, taking the minimum value. D：Deleterious (SIFT <=0.05); T：Tolerated (SIFT > 0.05)

#: Predict the effect of this variation on the protein sequence. The column contains two values, the first is the Polyphen2 score, the greater the value is "harmful ", indicating that the SNP is more likely to cause changes in protein structure or function; the second is D or P or B. (D: Probably damaging(>=0.957) ,P: Possibly damaging (0.453<=pp2<=0.956); B:Benign (pp2<=0.452))

##: Indicating the effect of this variation on the protein sequence. The comma before and after the MutationAssessor _score and MutationAssessor _pred：MutationAssessor _score is MutationAssessor initial scores, the larger the more likely to be "harmful ", indicating that the SNP is likely to cause changes in protein structure or function. MutationAssessor _pred is H、M、L or N (H: High; M:Medium; L: Low; N: Neutral).H and M are functional, L and N are non-functional.
